# Headache attributed to aeroplane travel: the first multicentric survey in a paediatric population affected by primary headaches

**DOI:** 10.1186/s10194-018-0939-y

**Published:** 2018-11-14

**Authors:** Debora De Carlo, Irene Toldo, Agnese Maria Tamborino, Barbara Bolzonella, Maria Giuseppina Ledda, Lucia Margari, Vincenzo Raieli, Margherita Santucci, Vittorio Sciruicchio, Angelo Vecchio, Sergio Zanini, Stefano Sartori, Michela Gatta, Alberto Verrotti, Pier Antonio Battistella

**Affiliations:** 1 Juvenile Headache Centre, University of Padua, via Giustiniani 3, 35128 Padua, Italy; 2 L’Aquila, Department of Pediatrics, San Salvatore Hospital, University of L’Aquila, L’Aquila, Italy; 3 Department of Child and Adolescent Neuropsychiatry, University Hospital of Cagliari, Cagliari, Italy; 4 Child Neuropsychiatry Unit, Department of Neuroscience and Sense Organs, University of “Aldo Moro” Bari, Bari, Italy; 5 Child Neuropsychiatry Unit, Di Cristina Hospital, ARNAS civico, Palermo, Italy; 6 Department of Child Neuropsychiatry, Università degli Studi di Bologna Scuola di Medicina e Chirurgia, Bologna, Italy; 7 Neurologic and Psychiatric Sciences Department, Ist Neurological Clinic, Bari, Italy; 8 Department of Child Neuropsychiatry, Università degli Studi di Palermo, Palermo, Italy; 9 IRCCS “La Nostra Famiglia - E. Medea”, Pasian di Prato, Udine, Italy

**Keywords:** Headache, Aeroplane travel, Airplane, Primary headaches, Children, Pediatric headache, ICHD-III

## Abstract

**Background:**

This multicentric survey investigates the prevalence and characteristics of Airplane Headache in children affected by primary headaches.

**Methods:**

Patients with symptoms of Airplane Headache were recruited from nine Italian Pediatric Headache Centres. Each patient was handed a structured questionnaire which met the ICHD-III criteria.

**Results:**

Among 320 children suffering from primary headaches who had flights during their lifetime, 15 (4.7%) had Airplane Headache, with mean age of 12.4 years. Most of the patients were females (80%). The headache was predominantly bilateral (80%) and localized to the frontal area (60%); it was mainly pulsating, and lasted less than 30 min in all cases. Accompanying symptoms were tearing, photophobia, phonophobia in most of the cases (73.3%). More than 30% of patients used medications to treat the attacks, with good results.

**Conclusion:**

Our study shows that Airplane Headache is not a rare disorder in children affected by primary headaches and highlights that its features in children are peculiar and differ from those described in adults. In children Airplane Headache prevails in females, is more often bilateral, has frequently accompanying symptoms and occurs at any time during the flight.

Further studies are needed to confirm the actual frequency of Airplane Headache in the general pediatric population not selected from specialized Headache Centres, with and without other concomitant headache condition, and to better clarify the clinical characteristics, pathophysiology and potential therapies.

## Background

Headache disorders in children and adolescents are common disabling problems with a significant impact on the quality of life of both children and parents [1, 2].

Airplane headache (AH) is a relatively rare headache disorder associated only with airplane travel; in particular pain begins during taking off or landing or both [3].

The first adult case of AH was reported in 2004 [4]. Its prevalence is unknown and the underlying pathophysiology is uncertain, although sinonasal barotrauma has been proposed [4–6].

In 2013 Mainardi et al. [7] collected clinical data of 75 patients with symptoms suggestive of AH and proposed provisional diagnostic criteria.

There has been a steadily increase in the number of reported cases in the following years: up to now, 275 adult cases have been described in the literature [7–20] and, recently, two systematic reviews have been published [21, 22]. Most of the known cases of AH are young males. In all cases, symptoms are highly stereotyped. The pain is typically reported as severe and may be described as jabbing, stabbing, or pulsatile in quality. It is usually unilateral and localized to fronto-orbital and fronto-parietal regions. The headache is short-lived, lasts less than 30 min, and occurs exclusively in relation to airplane travel (most frequently during airplane descent). Accompanying symptoms are usually absent.

So far only 5 cases within the pediatric age group have been described [23–25].

The aim of our study is to investigate the prevalence and characteristics of AH in a large group of children suffering from primary headaches, taking into account *experts’ opinion about the pediatric secondary headache diagnostic criteria of ICHD-III beta* [26].

## Patients and methods

This study is a multicentric pediatric cohort study. Patients with symptoms suggestive of AH were recruited from 9 Italian Pediatric Headache Centres.

During the study period, all patients were solicited to take part in the study. Following an explanation about the study purposes, each patient was handed a structured questionnaire, aimed at obtaining all the relevant information that could clinically distinguish this peculiar disorder. The questionnaire met the ICHD-III criteria [27].

The inclusion criteria were:

A. At least two episodes of headache fulfilling criterion C.
B. The patient is travelling by aeroplane.
C. Evidence of causation demonstrated by at least two of the following:
1. headache has developed during the aeroplane flight
2. either or both of the following:
  - headache has worsened in temporal relation to ascent following take-off and/or descent prior to landing of the aeroplane.
  - headache has spontaneously improved within 30 min after the ascent or descent of the aeroplane is completed.
3. headache is severe, with at least two of the following three characteristics:
  - unilateral location.
  - orbitofrontal location.
  - jabbing or stabbing quality.
D. Not better accounted for by another ICHD-3 diagnosis.
E. Age ≤ 18 years.

The structured questionnaire was composed of 32 questions and included two sections: medical history and details of nature of AH.

A total number of 727 children participated in this survey. The questionnaire was completed by 637 subjects.

Most of the cases were females (364 females, 273 males). The mean age of patients was 11.3 years (range 3.1–18.0).

## Results

### Age at onset

Among 320 patients who flew during their lifetime, only 15 (4.5%) had AH. Most of the cases were females (12 females, 3 males). The mean age at recruitment was 12.4 years (range 8.2–16.0); the mean age at headache onset was 7.2 years (range 3.1–11.1).

### History of coexistent primary headaches

A specific section of the questionnaire focused on the possible coexistence of a primary headache, according to the ICHD-III [27]. In the total study population (*n* = 637), the most commonly associated primary headaches were migraine (*n* = 456; 71.6%) and tension-type headache (*n* = 137; 21.5%). In 29 patients (4.5%) two different coexistent primary headaches could be diagnosed; no patient reported symptoms suggestive of cluster headache (Table 1).

**Table 1:** Classification of headaches in patients

Headache type	N	%
Migraine without aura	368	57.8
Episodic tension-type headache	82	12.9
Migraine with aura	74	11.6
Chronic tension-type headache	55	8.6
Migraine + tension-type headache	29	4.5
Other primary headaches	15	2.3
Chronic migraine	14	2.2
Total	637	100

The neurological examination was unremarkable. Basic biochemical and hematologic tests and brain magnetic resonance imaging revealed no abnormalities.

Twelve children (80%) with AH suffered also from migraine. Two patients (13.4%) with AH had also tension-type headache. One patient (6.7%) suffered from new daily persistent headache (Table 2).

**Table 2:** Concurrent primary headaches in AH patients

Headache type	N	%
Migraine with aura	7	46.7
Migraine without aura	5	33.3
Episodic tension-type headache	1	6.7
Chronic tension-type headache	1	6.7
New daily persistent headache	1	6.7
Total	15	100

### Upper respiratory tract disorders

A personal history for allergy was reported by 3 patients (20%), and for sinus infection by 3 patients (20%). No patients had symptoms and/or signs related to inflammatory sinus disorders following an ENT specialist evaluation, performed immediately after the attack.

### Clinical features of airplane headache

The clinical characteristics of AH were quite peculiar.

#### First episode

Seven children (46.7%) presented AH since the first flight experience.

Two patients (13.3%) flew 3–6 times per year, and seven patients (46.7%) flew less than one per year.

#### Consistency of attacks

In 7 children (46.7%) the same type of headache recurred consistently on separate flights; for those patients the pain started exclusively during landing in two, both during take-off and landing in three, during take-off in one and during cruising in one. The attacks occurred in more than 50% of flights only in one child (*n* = 1/15, 6.7%); for this patient the pain started only during take-off. The attacks occurred in less than 50% of flights in 3 patients (20%): exclusively during landing in two and also during cruising in one. In 4 cases (26.7%) the attacks were occasional and unpredictable. One patient reported the occasional occurrence of attacks during cruising only in the case of short-haul flights.

#### Timing

AH can occur at any time during the flight. More specifically, in three patients (20%) the attacks occurred exclusively during landing; in three patients (20%) AH started only during take-off. Five patient (33.5%) reported headache onset during cruising. In three patients (20%) AH started during both take-off and landing, and in one patient during cruising and landing (Table 3). No patients reported the possible difference in altitude between the arrival and departure airport as potential aggravating or trigger factors.

**Table 3:** Headache onset with respect to flight timing

	Female n (%)	Male n (%)
*Only during landing*	3 (20)	0
*Only during take-off*	1 (6.7)	2 (13.3)
*During cruising*	4 (26.7)	1 (6.7)
*During both landing and take-off*	3 (20)	0
*During both cruising and landing*	1 (6.7)	0
*Total*	12 (80)	3 (20)

#### Duration

The duration was less than 30 min in all patients.

#### Intensity

The pain intensity was defined as severe by 10 (66.7%), moderate by 3 (20%), and mild by 2 (13.3%) patients.

#### Quality of pain

Quality of headache was most frequently defined as pulsating (8 cases, 53.6%). Other definitions were: pressing in 4 (26.7%), tightening in 3 (20%) patients.

#### Localization of headache

In the majority of patients (12/15, 80%) the pain was bilateral. In the remaining 3 cases (20%) headache was unilateral; in these cases, the pain constantly recurred on the same side throughout the different attacks in two cases, while in one case (6.7%) the pain occurred on the opposite side in subsequent flights. The pain was more frequently localized to the frontal region (9/15, 60%). In three subjects (20%) the pain spread to the whole head.

#### Emotional impact

Only three children (20%) were concerned with the fear of suffering from a further attack and therefore were negatively predisposed for future flights. Among these subjects, 2 (13.3%) continued to fly with anxiety and/or worry; one (6.7%) decided to fly only if strictly necessary.

#### Accompanying symptoms

These were reported by 11 patients (73.3%): tearing, photophobia, phonophobia. No conjunctival injection, nausea, vomiting were reported.

#### Self-administered manoeuvres

Two patients (13.3%) performed one spontaneous maneuver in order to decrease the intensity of the attacks and to obtain some relief. The self-administered maneuvers were: pressure on the pain site and bracelet. One patient reported a reduction of pain intensity.

#### Pharmacological treatment

Ten patients (66.7%) did not take any drugs. The remaining 5 patients (3/5 females) used the following medications: acetaminophen (15 mg/kg), ibuprofen (10 mg/kg) and ketoprofen (1.5 mg/kg) were taken within few minutes after the headache onset, with rapid relief (Table 4).

**Table 4:** Demographic characteristics and clinical reports of the AH patients

Subject no	Gender	Age (year)	Duration of AH (minutes)	Intensity of pain	Quality of pain	Localization of the headache	Pharmacological treatment
1	F	12.6	30	5	Pressing	Temporal, bilateral	No
2	F	9.7	30	2	Pulsating	Frontal, unilateral	No
3	M	10.5	20	8	Pulsating	Frontal, bilateral	No
4	F	12.8	30	4	Pressing	Frontal, bilateral	No
5	F	8.2	20	9	Pulsating	Frontal, bilateral	Yes, acetaminophen
6	M	11	30	1	Pulsating	Frontal, bilateral	Yes, acetaminophen
7	F	15.5	20	8	Pressing	Frontal, bilateral	No
8	F	11	15	8	Pressing	Diffuse, bilateral	No
9	F	16	25	6	Pulsating	Frontal, bilateral	No
10	F	14	25	8	Tightening	Temporal, bilateral	No
11	F	12	20	9	Pulsating	Diffuse, bilateral	Yes, ketoprofen
12	F	16	30	8	Pulsating	Frontal, bilateral	No
13	F	15	30	9	Pulsating	Frontal, bilateral	No
14	M	9	30	7	Tightening	Diffuse, unilateral	Yes, acetaminophen
15	F	12.5	30	8	Tightening	Occipital, unilateral	Yes, ibuprofen

## Discussion

The present study was performed on a selected population affected by primary headaches referring to 9 Italian Pediatric Headache Centres and described in details the clinical features of 15 children suffering from brief headache during airplane travel. The clinical features of AH in our pediatric population were quite peculiar and differed from those described in adulthood [7–20].

Even though it is not possible to directly compare these data with those on AH adults recruited from the general population, it seems that AH features significantly differ between the adult and the child population in terms of several clinical features (intensity, distribution of pain, presence and type of accompanying symptoms, male to female ratio, etc). AH is strictly unilateral in the vast majority of adult cases, its phenotype resembles somehow cluster headache [7]. Moreover, only 20% of the pediatric patients presented a negative predisposition to future flights compared to 80% of the adult patients [7] and no one among the pediatric patients appeared to have restlessness or anxiety during the attack, maybe due to the less intensity of the pain. Children showed less frequently concern for subsequent airplane travel maybe for a reduced memory of the painful experience, for the lower intensity of pain and for a greater curiosity for future airplane travels. Moreover, differently from adults [7], no pediatric patients presented an easily recognizable postictal long-lasting mild headache phase after the AH acute attack.

All our patients suffered from at least one type of primary headache, as they were recruited in specialized pediatric Headache Centres. Only 20% of adults reported in the literature suffered from a concomitant primary headache [5–21]. Interestingly, we have found a relevant association between AH and migraine with aura. Baldacci et al. [10] reported a patient with AH also affected by migraine with aura: this primary headache is more rare compared to migraine without aura and episodic tension-type headache in the pediatric population. Therefore, the association between AH and migraine with aura does not seems to be incidental and deserves further epidemiological studies to better understand the etiopathogenetic mechanisms of this association.

In half of our AH cases, the first attack was concomitant with the first flight and in 46.7% of them occurred during each flight. In adult population the first AH during the first flight occured only in 14% of cases; that is a significant difference. It is likely that in the pediatric population, the headache attack during the flight warns the parents and therefore it is reported more frequently since the first attack.

The sex ratio was different for pediatric AH compared to adults [7–20] and was skewed toward females (80%) in our young patients. In adult population, the AH was unilateral and it was localized to the fronto-orbital area [7–20]. The quality of the headache was often defined as jabbing, stabbing and sharp [7–20]. Signs like tearing, conjunctival injection, nausea, vomiting, photophobia, phonophobia were observed rarely^7–20,^ while in our study population some of them (tearing, photophobia, phonophobia) were reported by the majority (73.3%) of patients.

Even though the pain was intense in most cases, only one third used analgesics both in children (33.3%) and in adults (38%) [7–20, 23–25].

Two patients performed one spontaneous maneuver to reduce the pain intensity and to obtain some relief, similar to the maneuvers carried out by patients with migraine, tension-type headache and cluster headache, as previously reported [25].

To the best of our knowledge, so far only 5 pediatric AH patients have been reported in the literature: in 2008 Mainardi et al. [23] described the first pediatric patient with AH; in 2010 Ipekdal et al. [24] described three pediatric AH cases aged between 12 and 14 years; the fifth case was reported in 2015 [25].

In the study of Ipekdal et al. [24], nasal mucosal inflammation, adenoidal and tonsillar hypertrophy and sinusitis were the pathophysiological mechanisms found to be responsible for AH. In all the 3 patients, AH has been solved by treating the underlying disease. In fact, they experienced other airplane travels and reported that they performed headache-free landings without any complication after the treatment.

In our study three children had a past history of sinus infections but none had any clinical evidence of an active sinus disorder during AH attacks.

Comparing the previously reported 5 cases with our series, we found that quality and intensity were similar, while site of pain was previously reported as unilateral [23–25], differently from our cases. A recent research article confirmed that headache caused by airplane travel is not necessarily unilateral, the description of headache may be not specific and the past history of any type of headache should be considered [26].

Given the peculiar clinical features of AH in the pediatric age compared to the adult population, more attention is needed towards this form of secondary headache, for better recognition even without the concomitance of other primary headaches: therefore the next edition of the ICHD should mention this entity also for the pediatric age.

## Conclusions

Our study shows that AH is not a rare disorder in children affected by primary headaches and highlights that features of AH in children are peculiar and differ from those described in adults.

The exact mechanism underlying AH remains unclear, but it has been suggested a multifactorial pathogenesis [28].

Further studies are needed in the general pediatric population to confirm the actual frequency of AH; in particular population-based studies might address the issue of analyzing AH incidence, considering a bigger sample not selected in specialized Headache Centres, with and without other concomitant headache condition, with a long-term follow-up evaluation.

## Abbreviations

AH: Airplane headache
ENT: Ear, nose and throat
*ICHD-III*: *International Classification of Headache disorders, 3rd edition*
